# Extent of substance abuse among patients with psychiatric disorders in Amman, Jordan

**DOI:** 10.1192/j.eurpsy.2024.246

**Published:** 2024-08-27

**Authors:** L. N. Abbasi, T. K. Daradkeh, M. ElWasify, S. Abassy

**Affiliations:** ^1^Medicine, Al-Balqa Applied University, Salt; ^2^Psychiatry, Jordan University of Science and Technology, Irbid, Jordan; ^3^Psychiatry, Mansoura University, Mansoura, Egypt; ^4^Medicine, Yarmouk University, Irbid, Jordan

## Abstract

**Introduction:**

The issue of substance abuse is increasingly being recogniszed as a significant global public health concern. In relation to its influence on the Arab world, scholarly investigation continues to be regarded as relatively constrained in scope.

**Objectives:**

The primary goal of the present study was to investigate the extent of substance abuse in a sample of patients with psychiatric disorders who attended a psychiatric clinic in Amman and analyse this in relation to demographic and clinical variables.

**Methods:**

In this prospective study, we investigated the prevalence, sociodemographic, and clinical characteristics of substance abuse among patients with psychiatric disorders who attended an outpatient private psychiatric clinic in Amman, Jordan between January and May 2023. Overall, 671 patients were enrolled. We compared demographic and clinical parameters of drug abuse and non-drug abuse patients.

**Results:**

Among the 671 patients included in the study, 48.0% (n=322) reported substance use, while 52.0% (n=349) did not. The mean age of the patients was 32.45±10.18 years. The sample included 546 male patients (81.4%). Among the participants, 388 (57.8%) were single, 360 (53.7%) held a bachelor’s degree or higher, 394 (58.7%) were employed, and 545 (81.2%) lived with their families. 506 (75.4%) were smokers, 110 (16.4%) reported having a medical illness, and 25% reported engaging in self-harm. The most prevalent psychiatric diagnosis was major depressive disorder, accounting for 30.6% of cases, followed by anxiety disorders. Additionally, 148 patients (22.1%) reported emotional abuse, 40 (6.0%) reported physical abuse, 57 (8.8%) reported sexual abuse, and 47 (7.0%) had a family history of substance use.

**Conclusions:**

Our research findings indicate that substance abuse is more prevalent among certain demographic groups, specifically young, unemployed, male patients with a low level of education and a family history of substance use. These findings highlight the need for further research in diverse settings and populations to better understand the scope and underlying factors contributing to substance abuse in Arab countries. Additionally, targeted interventions and prevention strategies should be developed to address the specific needs of these high-risk groups and reduce the burden of substance abuse in these communities.

**Disclosure of Interest:**

None Declared

